# Editorial: Comprehensive exploration of biomaterials and nanobiotechnology for tissue regeneration and organ reconstruction

**DOI:** 10.3389/fbioe.2025.1710115

**Published:** 2025-11-12

**Authors:** Yulin Jiang, Dongxu Ke

**Affiliations:** 1 School of Biomedical Engineering, Nanjing University Suzhou Campus, Suzhou, China; 2 Novaprint Therapeutics Suzhou Co., Ltd., Suzhou, China

**Keywords:** biomaterials, nanobiotechnology, tissue regeneration, organ reconstruction, patient-specific implant

## Introduction

Tissue or organ damage is a global health concern that imposes substantial medical and economic burdens on both individuals and healthcare systems (Niklason and Langer, 2001; Pockros et al., 2021). Traditional treatments, including autograft and organ transplantation, remain constrained by limitations such as donor shortages, risk of infection, and immune complications (Rana et al., 2015; Cozzi et al., 2017). Given the limitations of current therapies, tissue regeneration and organ reconstruction have emerged as promising strategies for replacing damaged tissues and organs, which bring both biomaterials and nanobiotechnologies to address pressing clinical needs (Loupy et al., 2025). One milestone of this field introduced the Integrated Tissue-Organ Printer (ITOP), a custom 3D bioprinting platform that could fabricate human-scale tissue constructs with precise geometry and mechanical stability, overcoming previous size and structural limitations (Kang et al., 2016). By utilizing nanostructured bio-inks and embedded microchannel networks for spatially controlled delivery of growth factors and vascular cues, the system advanced biomaterials-nanobiotechnology strategies toward functional tissue regeneration and organ reconstruction, which would revolutionize the modern healthcare in the future.

As a crucial part of this revolution, biomaterials serve as the foundation for tissue engineering by providing structural support, biochemical cues, and mechanical stability necessary for cell proliferation and differentiation (Kim et al., 2021). Natural biomaterials, such as collagen, hyaluronic acid, and fibrin, offer excellent bioactivity but face challenges related to mechanical strength and reproducibility (Ferreira et al., 2012; Zhu et al., 2017). While synthetic biomaterials, such as polycaprolactone (PCL), polylactic acid (PLA), and poly (lactic-co-glycolic acid) (PLGA), allow precise control over mechanical and degradation properties, yet may lack intrinsic bioactivity (Castañeda-Rodríguez et al., 2023; Patel et al., 2021; Siddiqui et al., 2018). Increasingly, composite biomaterials have been developed to combine the advantages of both natural and synthetic biomaterials, for example, hydroxyapatite-polymer composites for bone regeneration (Alkaron et al., 2024), PCL/gelatin fibers to mimic the vessels, and silk fibroin-carbon nanotube composites for neural regeneration (Rana et al., 2024).

Besides, nanotechnology has introduced innovative approaches to enhance tissue regeneration through the development of nanocarriers, nanostructured scaffolds, and bioactive nanoparticles (Kuru-Sümer et al., 2024). Nanoparticles such as gold, silica, and graphene oxide have been utilized for drug delivery, gene therapy, and imaging due to their high surface area and modifiable surface properties (Mitchell et al., 2021). Nanofibrous scaffolds fabricated via electrospinning also provide a biomimetic ECM-like structure that promotes cell adhesion, migration, and differentiation (Xie et al., 2020). Additionally, nanoscale coatings incorporating antimicrobial agents and growth factors have been developed to improve implant integration and reduce infection risks (Pugazhendhi et al., 2021).

The integration of biomaterials and nanobiotechnology has revolutionized tissue regeneration and organ reconstruction by enabling precise control over cellular environments (Gaharwar et al., 2020). Biomaterials provide scaffolds that mimic natural extracellular matrices, while nanobiotechnology enhances functionality through targeted drug delivery, improved mechanical properties, and bioactive signaling at the nanoscale (Chen et al., 2024; Jayabal, 2025). Innovations like smart nanomaterials and 3D bioprinting further advance personalized therapies, offering hope for complex organ repairs and regenerative medicine breakthroughs (Balasubramaniyam et al., 2025; Chandra et al., 2024). This synergy holds immense potential for treating injuries, degenerative diseases, and organ failure.

Additionally, the integration of biomaterials and nanobiotechnology has emerged as a transformative approach in tissue regeneration and organ reconstruction, addressing critical challenges in regenerative medicine. As a professor at Nanjing University Suzhou Campus (NUSC), our research focuses on “new engineering disciplines” and interdisciplinary collaboration. Leveraging its “equal standards, differentiated development” ethos, NUSC prioritizes cutting-edge research in biomedical materials, nanomedicine, and bioengineering, aligning with Suzhou’s industrial demands in biopharmaceuticals and advanced materials.

Despite its promise, the integration of biomaterials and nanobiotechnology faces many hurdles. Firstly, its biocompatibility must be rigorously tested to avoid immune rejection, and its biodegradation rates must align with tissue growth timelines. Secondly, long-term effects of nanoparticles, such as potential toxicity or unintended cellular interactions, require further study (Gupta et al., 2024). Thirdly, the cost and accessibility of these technologies raise concerns about equitable healthcare distribution. Finally, the manipulation of biological systems at the nanoscale demands careful regulation to balance innovation with safety (Elblová et al., 2025).

Although there are still many challenges, the integration of biomaterials and advanced nanobiotechnology facilitated with artificial intelligence (AI) and machine learning (ML) promises a future of patient-specific implants and organs. For example, 3D-printed scaffolds tailored to individual anatomies have already been used in craniofacial reconstruction (Hu et al., 2025). Additionally, a novel 3D bioprinting platform with AI and ML assisted real-time parameter adjustment for quality control has been created, which is a big step forward to the medical translation of bioprinting (Sergis et al., 2025; Tashman et al., 2022). As research progresses, collaboration across disciplines, such as material science, biology, and ethics, will be essential. The goal is not merely to replace tissues but to recreate their biological essence, heralding a new paradigm in regenerative medicine.

The seven papers collected for our special issues are all discussing about comprehensive exploration of biomaterials and nanobiotechnology for tissue regeneration and organ reconstruction (Amhare et al.; Chen et al.; Hlinkova et al.; Kian et al.; Ling et al.; Wang et al.; Zhang et al.). Hlinkova et al. demonstrated that electrospun polycaprolactone scaffolds with random versus aligned fiber orientations differentially regulated SH-SY5Y neuroblastoma cell behavior, revealing distinct pseudospheroid morphologies, gene expression profiles, and early network formation under high-density culture conditions. Chen et al. reported that Mn-MSN@Gel, a three-dimensional porous hydrogel with uniform elemental distribution, exhibits excellent mechanical properties, sustained ion release, and dual osteogenic-tenogenic differentiation promotion, effectively enhancing tendon-bone interface regeneration through anti-inflammatory and antioxidative mechanisms in a rat rotator cuff repair model. Ling et al. developed a multifunctional biomimetic GelMA-based periosteum functionalized with BMP-2-loaded M2 macrophage-derived exosomes, which synergistically promoted osteogenesis and immunomodulation. Amhare et al. highlighted the potential of nano-hydrogel-based scaffolds for osteochondral repair, emphasizing the importance of biomimetic design, hybrid polymer integration, and standardized evaluation to overcome current translational challenges and advance toward clinical application. Kian et al. demonstrated that decellularized walnut leaves (DWL) scaffolds retained bioactive components, exhibited biocompatibility, and enhanced wound healing in mice, making them promising for sustainable wound dressings. Wang et al. highlighted the recent advancements in 3D-printed biomaterials for osteoporosis treatment, emphasizing their advantages over conventional therapies in promoting osteogenesis, reducing inflammation, exhibiting antioxidant properties, and inhibiting osteoclast activity, while also addressing current limitations and future directions. Zhang et al. featured the transformative potential of vascularized composite allograft (VCA) transplantation in tracheal reconstruction, emphasizing advancements in revascularization techniques, regenerative medicine, and immune modulation strategies to overcome historical challenges and improve long-term outcomes for extensive tracheal defects. All these studies and reviews provide creative information in the field of biomaterials and nanobiotechnology and help advance its application for tissue regeneration and organ reconstruction in the future.
